# [Expression of Concern] Early anticoagulation therapy for severe burns complicated by inhalation injury in a rabbit model

**DOI:** 10.3892/mmr.2025.13691

**Published:** 2025-09-23

**Authors:** Zhong-Hua Fu, Guang-Hua Guo, Zhen-Fang Xiong, Xincheng Liao, Ming-Zhuo Liu, Jinhua Luo

Mol Med Rep 16: 7375–7381, 2017; DOI: 10.3892/mmr.2017.7537

Following the publication of this paper, it was drawn to the Editor's attention by a concerned reader that, regarding the histological images shown in Fig 1, the ‘Sham’ and the ‘Model’ data panels appeared to show areas of overlapping data with the ‘Union’ and the ‘Antithrombin’ panels respectively, albeit with very different zoom factors, such that data which were intended to show the results from differently performed experiments had apparently been derived from the same original sources. The authors were contacted by the Editorial Office to offer an explanation for these apparent anomalies in the presentation of the data in this paper; however, up to this time, no response from them has been forthcoming. Owing to the fact that the Editorial Office has been made aware of potential issues surrounding the scientific integrity of this paper, we are issuing an Expression of Concern to notify readers of this potential problem while the Editorial Office continues to investigate this matter further.

